# The Effect of Selected Dental Materials Used in Conservative Dentistry, Endodontics, Surgery, and Orthodontics as Well as during the Periodontal Treatment on the Redox Balance in the Oral Cavity

**DOI:** 10.3390/ijms21249684

**Published:** 2020-12-18

**Authors:** Izabela Zieniewska, Mateusz Maciejczyk, Anna Zalewska

**Affiliations:** 1 Doctoral Studies, Medical University of Bialystok, 24a M. Sklodowskiej-Curie Street, 15-274 Bialystok, Poland; 2 Department of Hygiene, Epidemiology and Ergonomics, Medical University of Bialystok, 15-022 Bialystok, Poland; mat.maciejczyk@gmail.com; 3 Experimental Dentistry Laboratory, Medical University of Bialystok, 24a M. Sklodowskiej-Curie Street, 15-274 Bialystok, Poland

**Keywords:** amalgam, antioxidants, dental resin composites, composites resins, endodontics treatment, glass-ionomer, orthodontics appliances, oxidative stress, periodontal treatment, redox balance, titanium implants, whitening

## Abstract

Oxidative stress (OS) is a redox homeostasis disorder that results in oxidation of cell components and thus disturbs cell metabolism. OS is induced by numerous internal as well as external factors. According to recent studies, dental treatment may also be one of them. The aim of our work was to assess the effect of dental treatment on the redox balance of the oral cavity. We reviewed literature available in PubMed, Medline, and Scopus databases, including the results from 2010 to 2020. Publications were searched according to the keywords: oxidative stress and dental monomers; oxidative stress and amalgam; oxidative stress and periodontitis, oxidative stress and braces, oxidative stress and titanium; oxidative stress and dental implants, oxidative stress and endodontics treatment, oxidative stress and dental treatment; and oxidative stress and dental composite. It was found that dental treatment with the use of composites, amalgams, glass-ionomers, materials for root canal filling/rinsing, orthodontic braces (made of various metal alloys), titanium implants, or whitening agents can disturb oral redox homeostasis by affecting the antioxidant barrier and increasing oxidative damage to salivary proteins, lipids, and DNA. Abnormal saliva secretion/composition was also observed in dental patients in the course of OS. It is suggested that the addition of antioxidants to dental materials or antioxidant therapy applied during dental treatment could protect the patient against harmful effects of OS in the oral cavity.

## 1. Introduction

Oxidative stress (OS) is defined as an imbalance between the production of oxygen (ROS) and nitrogen (RNS) free radicals and their neutralization by compounds called antioxidants [1]. Interestingly, ROS also act as signaling molecules involved in cell growth, proliferation and survival [2]. ROS/RNS are formed under the influence of external factors, such as ionizing radiation, ultraviolet radiation or ultrasound, as well as produced endogenously [3]. The main non-enzymatic, endogenous source of ROS/RNS in a cell is the mitochondrial respiratory chain. There are also enzymatic sources of ROS/RNS, including: xanthine oxidase (XO), cyclooxygenases (COX), lipoxygenases, myeloperoxidases (MPO), cytochrome P450 monooxygenase, uncoupled nitric oxide synthase (NOS), peroxidases and NADPH oxidase (NOX) [2]. The excess of ROS/RNS leads to the damage of cellular components such as proteins, lipids and nucleic acids as well as to cell death through apoptosis and necrosis [4,5].

Proteins are the main molecules attacked by ROS [6]. Highly reactive radicals cause multi-area damage to the protein side chain and backbone. Less reactive species demonstrate greater selectivity and damage smaller areas. Oxidation of proteins may cause increased hydrophilicity of the protein side chains, fragmentation of the side chains as well as the backbone, aggregation through covalent crosslinking or hydrophobic interactions, and protein conformation [7].

During protein peroxidation, cysteine and—to a lesser extent—methionine residues are oxidized. Usually hydrogen atoms are detached from the C-H or S-H bonds by the radical [7]. The separation of a hydrogen atom from carbon leads to the formation of a stabilized carbon-centered radical, which then reacts with other carbon-centered radicals or with O_2_ [8]. These reactions result in protein backbone fragmentation [8].

The side chains of amino acids are also subject to oxidative damage [9]. This mechanism usually leads to the formation of carbon-centered radicals. The amino acid side chains that contain sulfur are the most exposed to the oxidation process. Free amino acids are much less frequently damaged than the side chains, probably due to the fact that there are fewer of them than the side chains [7].

Lipid oxidation occurs in the presence of numerous oxidants, including peroxynitrite, hypochlorite, lipoxygenases, cyclooxygenase, cytochrome P450, and singlet oxygen [10]. Unsaturated lipids are particularly susceptible to oxidation. Under the influence of radical oxidants such as peroxyl radicals, lipid hydroperoxides are produced [11,12].

DNA oxidation usually results in breaks in the DNA thread, modification of bases and rupture of phosphodiester bonds. The consequence of these processes is oxidative damage to DNA. The marker of oxidative damage to nucleic acids is mainly 8-hydroxy-2′-deoxyguanosine (8-OHdG). Increased concentration of 8-OHdG was observed in the course of several systemic diseases caused by oxidative stress, such as cancer or diabetes. The formation of 8-OHdG is triggered by the product in the C8 position of the deoxyguanosine imidazole ring [11].

A special role in maintaining the redox balance is played by enzymatic and non-enzymatic antioxidant systems which protect cells against harmful effects of ROS [13,14]. Antioxidants, when present in low concentrations compared to the content of the oxidized substrate, are intended to inhibit the oxidation of this substrate [15]. The ROS/RNS neutralization process occurs in one or two stages. In the latter case, ROS/RNS are transformed into weak radicals that interact with another weak radical, leading to the formation of an inactive molecule. The most important enzymatic systems include: superoxide dismutase (SOD), catalase (CAT) and glutathione peroxidase (GPx), while the main non-enzymatic antioxidants are, inter alia: the glutathione system, albumins, lactoferrin, ascorbic acid (AS), uric acid and melatonin [16,17].

The oral cavity is the initial part of the digestive tract, a vital place due to the structures it contains, and further sections of the digestive system. It is a place exposed to numerous external factors inducing ROS/RNS production and OS development. Excessive production of ROS/RNS also occurs during the intake of food, drinks, and stimulants or as a result of dental treatment [18]. The cause of redox imbalance in the oral cavity may be pathological changes such as caries, gingivitis, periodontitis, pre-cancerous conditions and cancer, inflammation, and fungal infections [18]. Interestingly, more and more reports show that these diseases and their treatment disturbs the oral redox equilibrium. Evidence showed that the performed dental procedures, such as filling cavities, endodontic, periodontic, orthodontic, and surgical treatment, may increase the production of ROS/RNS, and thus the development of OS. Of course, it should be emphasized that the ROS/RNS generated during dental treatment also positively affects the oral cavity. They promote wound healing, stimulate the immune response, and facilitate the elimination of bacteria [18].

The aim of our study was a literature review of reports on the impact of dental, periodontal, orthodontic, and surgical treatment (in terms of titanium fixation and implants) on redox balance.

## 2. Materials and Methods

### 2.1. Search Strategy

A literature review was conducted from 2010 to July 2020. We used the databases of PubMed, Medline and Scopus, and analyzed only international literature in English. Publications were searched according to the entered key words: oxidative stress and dental monomers; oxidative stress and amalgam; oxidative stress and periodontitis, oxidative stress and braces, oxidative stress, and titanium; oxidative stress and dental implants, oxidative stress and endodontics treatment, oxidative stress and dental treatment; oxidative stress and dental composite.

### 2.2. Inclusion Criteria

Only the works meeting the following criteria were included in this paper:

1. Works on redox disorders related to dental treatment, dental fillings, dental monomers, endodontic treatment, titanium implants, treatment of periodontal diseases, whitening.
2. Results obtained from experiments participated by human subjects, as well as experimental works.
3. Publications in English only.
4. Clinical trials on a group of at least five individuals.

### 2.3. Exclusion Criteria

1. Works written in languages other than English.
2. Clinical trials on a group of fewer than five individuals.
3. Meta-analyzes.
4. Publications on the redox balance in the treatment of neurocranial diseases and cancer.
5. Publications referring to prosthetic treatment and treatment of functional disorders of the masticatory organ.
6. Case studies.
7. Among the publications based on human material we excluded those that covered subjects with systemic diseases.

## 3. Data Extraction

The titles and abstracts resulting from this search strategy were evaluated independently by two researchers (I. Z., A. Z). In case of doubt, the given publication was included or excluded after reading its entire text. Cohen’s kappa coefficient (κ) was used to measure interexaminer reliability (κ = 0.89). Each work was initially checked in the scope of its title, then abstract and the full text. When the studies met the inclusion criteria, they were included in this publication.

All works were evaluated in terms of methodology. Every publication had the following variables distinguished: authors, year of publication, study design, size of the study group, inclusion and exclusion criteria, duration of the study and study results.

## 4. Results

**Figure 1:**
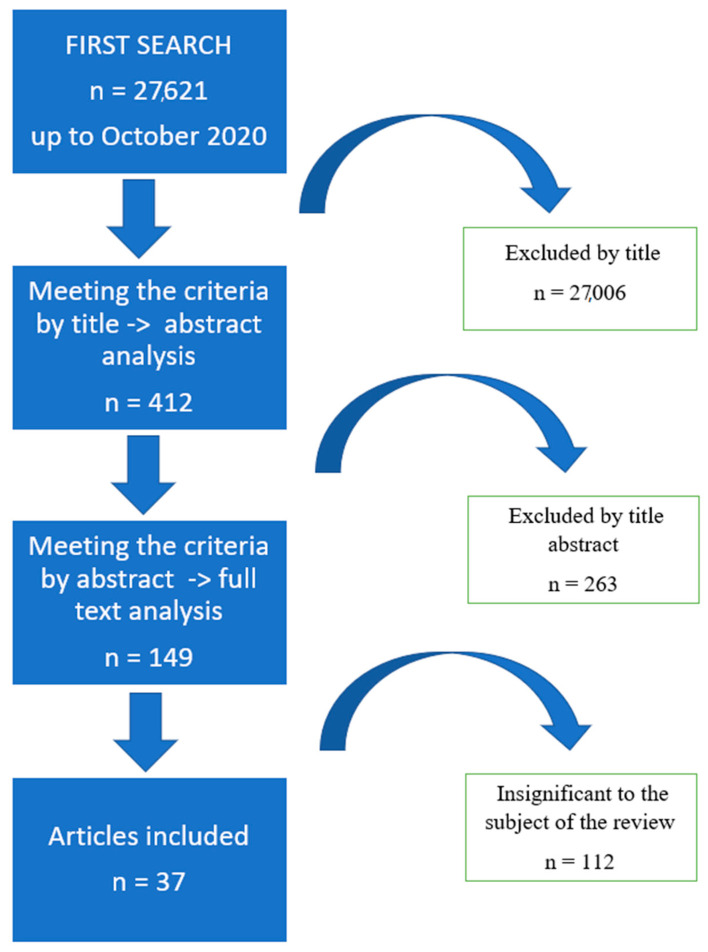
Flow chart of research methodology.

Out of approximately 27,600 publications, 37 were identified as meeting the inclusion and exclusion criteria. The literature review revealed 27,621 works from the MEDLINE (PubMed) library, of which 27,006 were excluded due to their title. A total of 412 summaries were read, 149 of which met the inclusion and exclusion criteria. Of the qualified articles, 112 proved irrelevant to the topic of our review. Therefore, 37 works were eventually included herein (Figure 1).

Research included the study are presented in Table 1.

## 5. Amalgam

The choice of filling materials is constantly increasing. Despite the dynamic development of cosmetic fillings, amalgam is still commonly used, which is due to its durability as well as low price, immediate availability and ease of use [22].

The influence of amalgam, both on the oral cavity and the entire body, has been discussed for a long time. The harmful effects of amalgam fillings are related to mercury contained in them. Studies demonstrated that mercury interferes with the metabolism of porphyrins which actively participate in numerous metabolic processes, including cellular respiration. The disruption of porphyrin metabolism may result in metabolic diseases, cancer or blood disorders, i.e., anemia or porphyrias [22,23]. Organic mercury, found in the form of methylmercury (MeHg), is considered particularly harmful [23]. Initial oxidative damage caused by MeHg in living organisms occurs as a result of its reaction with thiol (-SH) and/or selenol (-SeH) groups from endogenous molecules, leading to the formation of a very stable complex of type RSHgCH3 or RSeHgCH3 [58]. In proteins and enzymes containing thiol or selenol groups, the formation of S-Hg or Se-Hg bonds leads to the impairment of a given protein function [58,59,60,61,62] or entails the production of protein deposits rich in cysteine residues [63,64]. As mentioned above, due to high reactivity of MeHg to thiols and selenols, mercury occurs in living organisms in a form associated with these groups. In the human body, thiols are more common than selenols [65]; they can be found not only in compounds of low molecular weight (mainly cysteine and reduced glutathione), but also in proteins, whereas selenol groups are present only in a limited group of selenoproteins [66,67,68]. Considering the fact that both thiol and selenol groups are critical for the catalytic activity of numerous enzymes involved in antioxidant mechanisms and that MeHg reduces the activity of, inter alia, such enzymes (i.e., glucose-6- phosphate dehydrogenase [69], creatine kinase [60], glutathione reductase [70], glutathione peroxidase [59,71], thioredoxin reductase [10,72] there are grounds to postulate that this interaction disturbs the redox balance and results in increased production of ROS and RNS. However, studies by Cabaña-Muñoz et al. [23] revealed a significant boost in the activity of superoxide dismutase 1 (SOD-1) as well as reduced glutathione (GSH) concentration in hair samples collected from women who had amalgam fillings inserted 15 years prior to the study. According to the authors, stimulation of enzymatic antioxidant systems results from MeHg-induced elevated hydrogen peroxide (H_2_O_2_) concentration. It was shown that MeHg influences the mitochondrial electron transport chain (mainly at the level of complex II–III), which accelerates the formation of H_2_O_2_ [73]. The observed increased levels of the antioxidant barrier components as well as a positive correlation between mercury concentration and SOD-1 activity may suggest compensatory mechanisms to the chronic presence of mercury in women, aimed at counteracting Hg toxicity in individuals with amalgam fillings [23].

The results of the test for oxidative stress markers in subjects with amalgam fillings are contradictory, which is due to the test methods used, study material and the time that passed between the placement of a filling and the sampling. Al-Saleh et al. [22] observed abnormal concentrations of 8-hydroxy-D-guanosine (8-OHdG) and malondialdehyde (MDA) in the urine of children with amalgam fillings (no time passed from the filling insertion to the sampling was provided), and only 8-OHdG concentration was significantly correlated with the mercury content in the urine of these children. Mercury concentration in urine showed a clear dose-effect relationship with 8-OHdG level, mainly caused by long-term exposure to low Hg concentrations. The authors believe that oxidative damage to DNA of children is a result of exposure to Hg both from amalgam fillings and other sources. What is more, they argue that the presence of amalgam fillings does not exacerbate oxidative damage to DNA, but reduces the body’s ability to repair it, leading to a reduction in DNA repair products secreted into the urine compared to children without amalgam fillings. Studies indicated that 8-OHdG concentration in urine is not only a marker of oxidative modifications of the genetic material, but also reflects the efficiency of corrective mechanisms [74,75,76]. Interestingly, only the MDA concentration in urine was significantly correlated with the activity of urinary N-acetyl-β-D-glucosaminidase (NAG). It is worth mentioning that urinary NAG is used as a marker of renal tubular damage, especially in an environmental exposure to Hg [77]. The multiple regression model also demonstrated a statistically significant interaction between urinary Hg and MDA concentrations. This relationship suggests that exposure to Hg from amalgam fillings may result in renal tubular damage through oxidative stress (OS). In contrast, studies by Yildiz et al. [24] showed increased concentration of lipid peroxidation products (MDA) and no changes in the concentrations of oxidative markers of DNA damage in the plasma of patients 24 h after the placement of amalgam fillings. No correlation with Hg concentration was observed, which was consistent with the results of Daokar et al. [21]. These authors demonstrated a significant increase in MDA concentration in the saliva of patients who had had amalgam fillings inserted 2 weeks earlier.

In the study of Celik et al. [19] amalgam exhibited cytotoxic effect toward the HGF cell line, resulting in higher cell mortality and significantly higher total oxidant status (TOS) in freshly prepared samples compared to samples taken after 7 and 21 days. According to these authors, the decrease in cytotoxicity and TOS over time was caused by a considerable drop in the release of metal ions as the material hardened. Interestingly, amalgam had no significant effect on TOS and total antioxidant capacity (TAC) values compared to the control group. However, samples taken on day 7 and 21 demonstrated a considerably higher TAC compared to fresh samples. However, higher salivary TAC was observed in female and male children with two dental amalgam restorations (the time from the placement of the fillings to the collection of samples was not provided) compared to children who were caries-free and did not have any restorations [20]. Interestingly, females had higher TAC compared to males, which the authors claimed to be caused by hormonal changes typical of early adolescence and indicated more efficient antioxidant systems in girls in response to the increasing level of ROS.

In summary, mercury in amalgams is responsible for the redox imbalance in the system. MeHg modifies the mitochondrial electron transport chain function, which accelerates the formation of H_2_O_2_ [73]. A strengthening of the antioxidant barrier may suggest compensatory mechanisms to the chronic presence of mercury.

## 6. Glass-Ionomer Cement

Little is known about glass- ionomer-induced oxidative stress. It was demonstrated that metal ions released from glass- ionomer fillings are not toxic to cells and do not induce ROS formation, whereas polyacrylic acid [19] and fluorine ions released during the first phase of GI filling [19,78] have cytotoxic effects. During the first seven days, ROS production enhances, increasing TAC levels. The observed increase in TAC is an adaptive reaction of the cells that effectively prevents OS development, shifting the redox balance towards antioxidant reactions [19]. After 21 days, the level of TAC in the cell culture exposed to glass-ionomer filling did not differ from the control culture level, which most likely indicated that glass-ionomer filling was no longer cytotoxic to cells, and it was a sign of achieving redox balance.

## 7. Dental Resin Composites

In the present paragraph, we want to point out that we do not separate the cytotoxic effect of monomers from the potential toxic effect of dental resin composites once they were properly cross-linked. We take into account such differentiation in Table 1.

Dental resin composite materials are currently the most commonly applied materials for tooth reconstruction in conservative dentistry. In recent years, significant development in the dental materials of this group was observed. They are used especially for esthetic restorations in the front and back sections. It was documented that each resin-based material releases certain amounts of its components into the saliva. The most common of them are bisphenol-A-glycidyl methacrylate (Bis-GMA), urethane dimethacrylate (UDMA), triethylene glycol dimethacrylate (TEGDMA) and 2-hydroxyethyl methacrylate (HEMA). It is believed that HEMA is released in the largest amount from composite materials, and Bis-GMA—in the smallest, which is related to the size of the molecules as well as molecular weight of these monomers [79].

It is known that monomers released from composites contribute to genetic changes at the cellular level, and show cytotoxic effects [80]. It is generally believed that the cytotoxicity of these monomers might be ranked in the decreasing order: Bis-GMA, UDMA, TEGDMA and HEMA [79]. Moreover, studies showed that OS is responsible for monomer cytotoxicity [25,34], whereas silorane-based dental resin composites Hermes III, free of TEGDMA, HEMA and other monomers, does not lead to significant ROS/RNS production, and its cytotoxicity towards pulp cells is low compared to TEGDMA and HEMA-based dental resin composites [34].

A wide range of studies demonstrated that increased ROS/RNS production in resin-exposed cells occurs parallel and simultaneously to the depletion of reduced glutathione resources [27,28,46]. The glutathione system is the most important cellular detoxification mechanism against ROS activity [81]. GSH is a tripeptide (--glutamylcysteinylglycine) with a molecular weight of 307 kDa. GSH enables the removal of free radicals from the body either directly or through the reactions catalyzed by glutathione peroxidase and other peroxidases, thus neutralizing hydrogen peroxide as well as nitrogen peroxides [82]. GSH reacts with electrophiles, creating less reactive conjugates. Interestingly, decreased concentration of GSH in human dental pulp cells (HDPCs) exposed to resin-based composites is not accompanied by increased level of oxidized glutathione (GSSG), which indicates that reduced amount of GSH resources does not result from its oxidation [25]. Studies have shown that carbonyl groups of methacrylates, adjacent to the double carbon-carbon bond, act as electron withdrawing groups. Consequently, carbon in the beta position of the double bond, as a positive charge, can react with nucleophilic centers of amine or thiol groups in small molecules such as GSH in the Michael-type addition reaction [25]. Indeed, Samuelsen et al. [83] observed spontaneous formation of a complex between HEMA and GSH. They concluded that exposure to HEMA leads to a drop in cellular GSH level, probably due to the formation of a complex with HEMA. Nocca et al. [84] found that the kinetics of the formation of GSH-methacrylate adducts depended on the reaction environment. The rate of adduct formation was different when the reaction occurred in human fibroblasts or erythrocytes than when the reaction environment was a mixture of methacrylates and GSH, which suggests that the reaction is strongly correlated with glutathione S-transferase. Studies by Schneider et al. [26] proved that the drop of GSH concentration in pulp cells caused by TEGDMA after 2 h from exposure was comparable to the decrease in GSH concentration caused by Bis-GMA and UDMA after 48 h. Interestingly, the TEGDMA-induced decrease in GSH concentration was a reversible process, and after the exposure to Bis-GMA and UDMA the treatment further decreased. Evidence showed that Bis-GMA and UDMA significantly reduce cystine uptake, while TEGDMA has the opposite effect. The so-called system x_c_^-^ is responsible for the transport of cystine into dental pulp cells in which it is transformed into cysteine that is used for the production of GSH [35]. According to the authors, this explains the observed phenomenon of oxidative stress in the pulp cells exposed to Bis-GMA and UDMA, and its absence in the cells treated with TEGDMA. GSH directs the expression of enzymatic antioxidants that are exposed in cells to monomers, including HEMA. It was demonstrated that HEMA reduces the activity of glutathione peroxidase GPx1/2, but in the presence of L-buthionine sulfoximine (BSO), which inhibits GSH synthesis, this reduction is more intense. Moreover, significantly elevated ROS generation as well as increased catalase (CAT) activity are observed [36,85]. It is noteworthy that the inhibition of GSH synthesis by BSO in cells not treated with monomers boosts GPx1 activity as a result of increased H_2_O_2_ concentration due to GSH reduction. However, it seems that in cells not treated with monomers the concentration of H_2_O_2_ is low, because no increase in CAT concentration was observed. It was proven that GPx1/2 regulates lower H_2_O_2_ level, whereas CAT expression is induced by higher H_2_O_2_ concentrations observed in HEMA-treated cells [85].

Interestingly, the use of polyester film which reduces the occurrence of oxygen-inhibited free radical polymerization of dental polymers, results in increased monomer conversion and inhibited ROS production in pulp cells exposed to monomers [34].

According to Diomede et al. [29], a significant, spontaneously irreversible increase in ROS concentration in HDPSCs treated with HEMA after 24 h of exposure is not connected with GSH depletion, but results from mitochondrial dysfunction. Confocal microscopy of cells exposed to HEMA indicates 1000 times higher signal from ROS-sensitive indicator in the mitochondrial area compared to control cells. These differences are most likely due to HEMA-induced dysfunction of the respiratory chain dysregulating the oxidative phosphorylation process. The results of Diomede et al. [29] are consistent with the work of Jiao et al. [25,32] who observed morphological disorders of mitochondria, including their elongation and cristae derangements, depolarization of mitochondrial membranes, reduction of oxidative phosphorylation rate in cells exposed to HEMA and decrease of ATP production. Interestingly, mitochondrial dysfunction induced by monomers exacerbates ROS-induced damage to the dental pulp cells through bioenergetic failure and the internal mitochondrial apoptosis pathway [25,86]. The mitochondrial chain, located in the internal mitochondrial membrane, consists of four enzymatic complexes transporting electrons (CI-CIV) and the ATP synthase enzyme. [87]. It is still unknown which of the complexes is damaged in the course of exposure to monomers. A recent study showed CI as the most important toxicity target of TEGDMA [88]. However, in HEMA-exposed cells, the suppression of all four complexes, either by nuclear-encoded mitochondria or mtDNA-encoded transcription, was observed [89]. These discrepancies are certainly due to the method of mitochondrial isolation as well as the type of resin monomer used. It was demonstrated that although both monomers exhibit a similar toxicity mechanism, cells react differently to various resins by differential induction of the cell death (e.g., only HEMA induces autophagy in human gingival fibroblasts) [33]. Jiao et al. [25] demonstrated that NAC reduces monomer-induced oxidative stress in human pulp cells. Styllou et al. [90] found that the addition of NAC to human fibroblasts exposed to TEGDMA and HEMA significantly minimizes double strand breaks and restores cell nucleus integrity. The authors believe that the protective effect of NAC results from the ability of this molecule to neutralize ROS and replenish GSH deficiencies as well as from the direct reaction of NAC with methacrylate groups of monomers in the Michael addition. The presence of ascorbic acid (AS) counteracts the oxidative effects of HEMA, restoring ROS concentration to the level observed in the control group. AS seems to mimic the effect of NAC, because both molecules neutralize HEMA-induced ROS growth in the cytosol, with only AS being able to completely prevent excessive ROS production in mitochondria [29].

Cells have numerous mechanisms to counteract OS. One of them is the activation of the transcription factor: nuclear factor erythroid 2-related factor 2 (Nrf2)-dependent signaling pathway. Under equilibrium conditions, redox Nrf2 is synthesized and quickly degraded. In a situation of increased ROS production, Nrf2 is stabilized and transferred to the cell nucleus in which it activates the transcription of numerous genes by binding the promoter to antioxidant responsive elements (ARE) [91]. The most important genes activated by Nrf2 include heme oxygenase (HO-1), NAD(P)H quinone dehydrogenase 1 (NQO1) and superoxide dismutase 1 (SOD1). These enzymes play an important role in regulating the redox balance and counteracting monomer toxicity. Durante et al. [92] and Gozzelino et al. [93] believe that antioxidant bilirubin, resulting from the catalytic activity of HO-1, is an essential next link in strengthening the antioxidant barrier when exposed to monomers, and directly balance the cellular redox environment. The stimulation of Nrf2 signaling cascade was observed in HEMA-exposed RAW264.7 mouse macrophages [85,94], human fibroblasts [30,31] and the immortalized human oral keratinocyte cell line (OKF6/TERT-2) [31]. Interestingly, in the latter cell line it was noted that Nrf2 has a potential to enhance its own transcription through a positive feedback loop. Perduns et al. [31] observed that even “non-toxic” HEMA concentrations (0.5 mM) induce the expression of genes of antioxidant enzymes HO-1, NQO1 and SOD1 in human fibroblast and keratinocyte cells. Ramezani et al. [20] showed that dental resin composites significantly enhanced total antioxidant capacity (TAC) in saliva, as TAC level was higher than that of amalgam fillings [31]. Celik et al. [19] obtained increased TAC in gingival fibroblasts (HGFCs) after 7 days of exposure to dental resin composites as well as weakening of the antioxidant barrier on day 21, which was caused by a rapid boost of ROS production from unbound and non-polymerized monomers. The level of HEMA higher than 5 mM enhances ROS production to such an extent that we can observe the occurrence of ROS-induced gene expression disorders associated with inflammation (NF- κB, TNF-α, IL-6, IL-8) and remodeling of the extracellular matrix (COL1A, COL4A, metalloproteinase 9—MMP-9, tissue inhibitor of metalloproteinase 1—TIMP1) [19]. Indeed, studies have revealed that monomer-induced OS acts as a signal for the activation of the pathways controlling cell survival and death, although the exact mechanism of this phenomenon is unknown. It was observed that HEMA significantly increases the number of cells in late phase of apoptosis and necrosis [85]. An additional supply of cysteine, an amino acid essential for the synthesis of GSH by 2-oxothiazolidine-4-carboxylate (OTC), considerably reduces the number of apoptotic and necrotic cells. These observations suggest that HEMA-induced cell death is due to, inter alia, GSH deficiency [85]. These observations are consistent with the study by Lee et al. [95] in which the authors observed increased cell survival after the supplementation of 10 mM NAC. NAC activates GSH reductase, which leads to an increase in GSH concentration.

Yildiz et al. [24] showed that Bis-GMA and TEGDMA, and Jiao et al. [32]—that HEMA and TEGDMA significantly increase MDA concentration in human dental pulp cells. It is noteworthy that the measurement of MDA content at various times after the placement of the dental resin composites revealed its linear increase with the time passed from the material application [37]. Daokar et al. [21] observed that the salivary MDA concentration two weeks after the placement of dental resin composites, the authors did not demonstrate any differences in salivary MDA concentration after the placement of composite and glass-ionomer fillings. In another study, which evaluated TEGDMA and Bis-GMA in dental pulp cells, the increase of 8-OHdG post-application content was noted, and ROS production was correlated with DNA oxidation [24]. Interestingly, the concentration of 8-OHdG was significantly higher in samples after the application of the dental resin composites compared to the amalgam filling group. These observations prove that monomers cause higher oxidation and transformation of the genetic material of dental pulp cells compared to amalgam fillings. It was shown that resin monomers, such as HEMA, lead to the formation of double strand breaks [96,97,98]. Anteisson et al. [99] demonstrated that HEMA-induced genome damage results in the stimulation of ataxia telangiectasia mutated (ATM) gene and other cell cycle checkpoints, which leads to the activation of kinase signaling networks that impede the progression of the cell cycle and simultaneously activate DNA repair pathways. Similarly, Blasiak et al. [38] found that resin monomers increase the expression of 8-hydroxyguanine in DNA—hydrolase 1, the main enzyme for repairing 8-oxoG damage by trimming alkalis.

The adverse effects of dental resin composites can be attributed to monomers’ presence and the influence of the composite once they were properly cross-linked. Glutathione depletion is responsible for GMA, HEMA, TEGDMA-induced OS. Moreover, HEMA and TEGDMA were shown to disrupt the mitochondrial respiratory chain’s functioning and disrupt the oxidative phosphorylation process, which contributes to the exacerbation of ROS-induced apoptosis to the dental pulp cells. Interestingly, although dental resin composites increase TAC higher than amalgam fillings, unfortunately, monomers cause higher oxidation and transformation of dental pulp cells’ genetic material than amalgam fillings.

## 8. Composite Resins

Low-viscosity resins are commonly used in restorative dentistry as binding agents connecting dental materials with dental tissue. The inclusion of iodine salts in dental bonding systems was found to be very interesting because they can act as catalysts, reducing activation energy. Due to their ionic nature, they participate in the polymerization of hydrophilic monomers and thus increase the polymerization reaction capacity and percentage of monomer conversion rate. Ferrúa et al. [60] found higher activity of superoxide dismutase (SOD) in 10 s of polymerization vs 20 s, which indicates a slight response of mouse fibroblast cells (NIH/3T3), most probably connected with increased monomer release in the initial phase of polymerization. Significant reduction of lipid peroxidation and oxidation of disulfide groups, after incorporation of iodine salts into bonding systems, was—according to the authors—associated with the inhibition of ROS producing enzymes and proved their protective action towards cell membranes.

Initiators are an essential component of light-hardened composites and dental adhesives. Currently, visible-light photoinitiators, such as phenylbis(2,4,6-trimethylbenzoyl) phosphine oxide (BAPO) and diphenyl(2,4,6-trimethylbenzoyl) phosphine oxide (TPO), are used. Both of these photoinitiators belong to the group of Norrish type I photoinitiators and unlike camphorquinone (CQ) they do not generate ROS. Popal et al. [100] found that both BAPO and TPO used in micromole concentrations do not increase ROS/RNS production in human keratinocytes and V79 fibroblasts during 90 min of exposure. Interestingly, the concentration of ROS/RNS in cells treated with the said photoinitiators was lower compared to the control cells. It is probable that aromatic and phosphine oxide groups serve as electron donors that compete with the fluorescent dye DCFH_2_ used in the ROS detection method. In the absence of significant changes in ROS/RNS concentration, the alterations in mRNA expression of enzymatic antioxidants in the keratinocyte culture exposed to BAPO and, to a lesser extent, to TPO prove that these cells may be subjected to OS. Within 24 h, BAPO induced a significant increase in HO-1 mRNA and quinone oxidoreductase (NQO1), and TPO only triggered a significant increase in HO-1 mRNA. Yoshino et al. [101] found that long-term irradiation of the human aortic smooth muscle cells (ACBR1716) with blue light (from a quartz-tungsten-halogen lamp) induced the production of H_2_O_2_ and hydroxy radical (HO^.^). As a result, increased peroxidation of lipid membrane as well as MDA production were observed. They also proved that treatment with NAC could reduce ROS production and cytotoxicity induced by blue light irradiation. Oktay et al. [102] observed markedly increased TOS in the absence of significant changes in TAS level in rat aorta cells irradiated with blue light (400–520 nm, 1200 mV/cm^2^) vs not irradiated cells. Based on the results of these studies, it is difficult to assess whether the rat aorta cells were subjected to OS or whether the antioxidant barrier was efficient enough to balance the emerging ROS/RNS.

Although BAPO and TPO show a dose-dependent cytotoxic effect, unlike CQ, they do not increase the intracellular production of ROS /RNS. Interestingly, dental resin curing blue light alone induces increased peroxidation of membrane lipids.

## 9. Endodontic Treatment

The complicated morphology of the root canal system of teeth makes instrumentation challenging. One of the most important elements of root canal treatment is the use of irrigating solutions which are designed to neutralize microorganisms as well as dissolve and remove tissue residues, including the smear layer. These solutions come into direct contact with periapical tissues, particularly in teeth with periapical lesions in which the identification of the anatomical apex is difficult, and the physiological opening is virtually non-existent due to periapical microresorption. The most commonly used rinsing solutions include the main ones: sodium hypochlorite (NaOCl) and chlorhexidine (CHX) as well as auxiliary solutions: ethylenediaminetetraacetic acid (EDTA) and citric acid. Botton et al. [103] observed that 2% CHX and 6% citric acid did not cause lipid peroxidation, regardless of the exposure time of human peripheral blood mononuclear cells (PBMCs). The 72-h exposure of PBMCs to both 1% and 2.5% NaOCl resulted in increased lipid peroxidation, which suggests that prolonged contact of the cells with the rinsing solution may result in OS and, consequently, disturbed integrity of cell membranes due to the destruction of the lipid bilayer [104]. The solution of 17% EDTA boosted the process of lipid peroxidation only after 24 h of exposure of the cells, which was not maintained after 72 h (Botton et al. [103]), co Saghiri et al. [104] is the adaptation of cells to environmental conditions. The genetic material appears to be more susceptible to the damaging effects of the rinsing agents, as increased oxidative DNA modifications were observed after both 24 and 72 h of exposure of PBMCs to all the flushing solutions. A similar genotoxic effect was observed by evaluating combinations of major rinsing solutions with auxiliaries after both 24 and 72 h of exposure. Soares et al. [105] observed no DNA damage due to NaOCl (1.25%, 2.5%, 5%), 17% EDTA or citric acid (10.5% and 21%); however, the exposure time in their study was only 3 h.

The effectiveness of the described disinfectants is supported by the so-called photodynamic antibacterial chemotherapy (PACT). PACT uses a light source of the narrow-band wavelength that activates rinsing chemicals [106]. A by-product of this reaction are oxygen free radicals such as ozone and H_2_O_2_, which on the one hand, help to eliminate infections in the root canal but, on the other, may contribute to cell death.

The final stage of endodontic treatment is filling the canal system. Different materials are chosen depending on whether the roots of primary or permanent teeth are filled. However, the filling materials should not exhibit any cytotoxic effect, but should have antibacterial properties in order to prevent reinfection. In the study by Pires et al. [107] iodoform-based pastes used to fill the root canals of deciduous teeth induced a significant increase in ROS already after 24-h exposure of PBMC cells. Interestingly, after 72 h of exposure to iodoform preparations, further significant increase in ROS concentration in the said cells was not accompanied by intensified lipid peroxidation, which—according to the authors—was due to an efficient antioxidant barrier capable of restoring the redox balance. It should be noted that pastes containing chlorhexidine induced significantly higher ROS production compared to iodoform pastes with neomycin sulfate + bacitracin as well as rifamycin SV sodium + 21 prednisolone acetate. According to Barbin et al. [108], redox imbalance induced by CHX was related to parachloroaniline and ROS-byproducts of the 2% CHX aqueous solution. Parachloroaniline has a high potential to cause oxidative DNA damage [81]. Calcium hydroxide-based pastes triggered the highest ROS increase after 24 h of exposure. After 72 h, only the paste with zinc oxide induced further significant increase in ROS production in the PBMC culture. In the TBARS assay, calcium hydroxide pastes demonstrated a similar increase in lipid peroxidation within 24 h, whereas no calcium hydroxide paste caused oxidative damage to the lipids after 72 h of exposure. All calcium hydroxide pastes and just one of the iodoform pastes (CHX) showed the ability to induce oxidative DNA damage, regardless of the exposure time.

Among the materials applied in endodontics to treat permanent dentition, root canal sealers are sealing materials in most methods based on the use of gutta-percha for canal filling. The most common include AH-Plus pastes (Paste A: bisphenol-A, bisphenol-F, calcium tungstate, zirconium dioxide, silicon monoxide, ferric oxide; Paste B: dibezyl diamine, aminoadamantane, tricyclodecane diamine, calcium tungstate, zirconium dioxide, silicon monoxide, silicone oil), MTA-Fillapex (base paste: salicylate resin, natural resin, calcium tungstate, silica nanoparticles, dyes; catalyst paste: diluted resin, mineral trioxide aggregate, silica nanoparticles, dyes) and materials applied in regenerative endodontics: MTA-Angelus (SiO_2_, K_2_O, Al_2_O_3_, Na_2_O, Fe_2_O_3_, SO_3_, CaO, Bi_2_O_3_, MgO and insoluble deposits of CaO, KSO_4_, NaSO_4_ and crystalline silica), Biodentine (tricalcium silicate, zirconium dioxide, calcium carbonate, calcium chloride, polymer) and BioRoot (tricalcium silicate, zirconium dioxide, calcium chloride, polymer). The results of the study by Victoria-Escandell et al. [109] demonstrated that MTA-Angelus did not induce OS w HDPSCs after 24 h of incubation, while AH-Plus and MTA-Fillapex increased protein carbonyl concentration, which is consistent with the results of Kim et al. [110]. Chang et al. [111] demonstrated that MTA-Angelus is capable of increasing ROS production, but the simultaneously activated antioxidant barrier is strong enough to maintain the redox balance. In HDPSC cells treated with AH-Plus and MTA-Fillapex for 24 h, both CAT and SOD were down-regulated compared to the control and MTA-Angelus-treated cells [109]. Under the conditions of low ROS production, which—according to the authors—occurs during the exposure to MTA-Angelus, cells can respond by activating Nrf2 expression and then the target genes of antioxidant enzymes, e.g., CAT or SOD. In the state of OS that occurs in case of AH-Plus and MTA-Fillapex, the Nrf2 pathway is suppressed and the activity of antioxidant enzymes is decreased. No significant differences were observed in the gene expression of CAT, SOD, glutathione synthase in the human dental pulp cells treated with Biodentine and BioRoot [112]. The authors did not examine oxidative stress markers; therefore, it is difficult to assess the occurrence and possible severity of OS.

Most endodontic materials generate increased amounts of ROS, leading to the development of the OS. However, it should be emphasized that biocompatible materials used in the form of sealers seem to be the most favorable from the point of view of redox balance.

## 10. Orthodontic Braces

Treatment with braces is becoming more and more popular all over the world. Fixed braces are made of various steel alloys, titanium or Ni-Ti. It was demonstrated that some metals included in the alloys of which braces are made (such as iron, chromium, copper, vanadium) are directly responsible for increased ROS production [60,61], while such metals as cadmium, mercury, nickel or lead generate ROS via the indirect mechanism [60]. Interestingly, nickel increases the activity of intracellular lactate dehydrogenase, which disturbs the redox balance and stimulates apoptosis in human oral epithelium cells [113]. Moreover, it is suspected that titanium elements of dental braces are deprived of their superficial layer of titanium oxide as a result of mechanical friction during treatment, which leads to corrosion and the release of metallic particles into the oral cavity environment. The significance of this phenomenon for the redox balance is explained below. The available research results concerning the influence of orthodontic braces on the oral redox balance are contradictory and, unfortunately, limited to only several scientific studies. Özcan et al. [43] suggest that orthodontic treatment, including the use of braces, does not increase the oxidative damage observed in the saliva and gingival fluid. Kovac et al. [61] observed that both ROS and the ratio of ROS to antioxidants in the blood clearly increased within a short period of time after the fitting of braces (24 h) compared to the control group. These levels normalized 7 days after the beginning of orthodontic treatment. Buczko et al. [44] found significantly elevated levels of TBARS and TOS in the unstimulated and stimulated saliva one week after orthodontic treatment (nickel-chromium archwires) and no OS biomarkers in the saliva of orthodontically treated patients within 24 weeks of wearing dental braces. This initial severity of OS is associated with intensified nickel release, as confirmed by a positive correlation between nickel and TOS concentrations in both unstimulated and stimulated saliva in week 1 of the use of braces. In the 24th week, the salivary nickel concentration was comparable to its content before orthodontic treatment. The authors emphasize that the treated patients maintained perfect oral hygiene and showed no gingivitis. Oral hygiene and periodontal health are crucial for the salivary redox balance. Similar results were obtained by Portelli et al. [42], with measurements taken before as well as 5 and 10 weeks after the start of treatment. The researchers claimed that multi-bracket archwires with vestibular appliances do not induce OS during the first 10 weeks of treatment, which is associated with the fact that this type of braces reduces the forces exerted on the teeth and allows for good oral hygiene. On the other hand, the results of Spalj et al. [40] revealed that nickel-titanium standard braces generated the strongest, while stainless steel and titanium-molybdenum ones the least intensive OS in the L929 mouse fibroblast cell line. The use of copper-nickel-titanium and rhodium-coated nickel-titanium orthodontic appliances resulted in increased production of 8-OHdG, which was lower; however, than in the case of standard nickel-titanium archwires. Cobalt-chromium archwires triggered moderate 8-OHdG production, which was most probably connected with the suppression of mitochondrial activity in the aforementioned cell line. Similar results were obtained by Buljan et al. [41], but the authors observed that the strongest OS expressed in 8-OHdG concentration was induced by polymer brackets, which most likely resulted from the presence of polyether polyol. Moreover, the researchers demonstrated that not only the mentioned polymers, but also zirconium dioxide and synthetic sapphire used in the production of elements of aesthetic braces were not indifferent to salivary redox balance, as expressed by increased 8-OHdG concentration in the L929 murine fibroblast cell line.

All orthodontic appliances induce OS. Its intensity depends on the time and the metal alloys used. Initial severity of OS is believed to be associated with intensified nickel release.

## 11. Fixations and Dental Implants

Titanium and its alloys are commonly applied for the production of medical implants. They are widely used in maxillofacial surgery and orthopedics as bone fixations, joint prostheses, dental implants, and other devices used in reconstructive surgery. Implants made of titanium and its alloys are very popular due to their good mechanical properties, corrosion resistance and biocompatibility. Their higher biocompatibility in human tissues, compared to other metallic materials, is related to the presence of an inactive layer of titanium dioxide (TiO_2_) on the implant surface. However, clinical and experimental studies showed that ions and particles of titanium and its alloys are found in peri-implantation tissues, blood and distant organs after the placement of the implants. Gholinejad et al. [114] observed that titanium dioxide nanoparticles were internalized to HUVECs and induced intracellular ROS production as well as cell membrane oxidative modifications. Borys et al. [48,50] demonstrated that exposure to the titanium alloy Ti-6Al-4V stimulated antioxidative mechanisms in the periosteal cells covering titanium implants in the jaw and mandible. However, this defensive reaction was insufficient as it did not prevent oxidative damage to proteins (↑protein carbonyl) and lipids (↑MDA) in the periosteum-like tissue in the area adjacent to the implants [46]. The results obtained by the authors indicate the persistence of OS phenomena around the fixations of maxillary bones regardless of the time that passed from the surgery of facial bone defects, but these phenomena were not accompanied by any clinical symptoms [48]. Moreover, the authors indicate that the production of ROS results, among others, from mitochondrial dysfunction (decreased activity of complex I and citrate synthase) [46] as well as increased activity of NADPH oxidase and xanthine oxidase [49]. Mitochondrial function disorders in muscle cells and their morphological changes (swelling and vacuolization) were confirmed by the studies of Wang et al. and Pereira et al. [115,116]. Furthermore, Pereira et al. [115] showed that exposure of rat liver mitochondria to TiNPs (titanium nanoparticles) + AgNPs (silver nanoparticles) lowered the respiratory control ratio, which resulted in reduced oxidative phosphorylation efficiency. 21-day exposure of mitochondria to both types of nanoparticles maintained the increased ROS levels and depleted the endogenous antioxidant system. AgNPs and TiNPs acted synergistically: the intensity of the toxic effect on the mitochondrial redox balance was more significant in the presence of both types of nanoparticles. Borys et al. [49] also observed a positive relationship between the production of ROS/RNS and the concentration of titanium, aluminum and vanadium in tissues surrounding the titanium implants, which is explained by the observations of Shanbhag et al. [117] and Mouthuy et al. [118]. These authors demonstrated that the said ions released from the implants stimulate macrophages and osteoclasts to increase ROS and RNS production, and that ROS/RNS boost the release of metal ions from the implant surface through ROS/RNS-induced corrosion. According to these researchers, these observations highlight the need to improve the quality of the applied jawbone fixations by increasing the passive TiO_2_ layer thickness in miniplates and screws in the process of hard anodizing, or search for other materials, preferably biodegradable in human tissues, for the production of dental fixations.

In recent years, we witnessed rapid development of implantology as a field of dentistry. Implantation is a more and more common method of filling in missing teeth [63]. The maintenance of an implant in the oral cavity depends on numerous factors. Peri-implantitis has an inflammatory etiology, therefore the effect of OS on the phenomenon of implant rejection is sought. However, the results of these studies are contradictory, depending on the type of the examined material or clinical situation. At the beginning, it is worth mentioning that ROS act as mediators of cytoskeletal remodeling and cell proliferation, while too low intracellular ROS concentration was found to slow down cell adhesion and proliferation. The results of Wei et al. [45] demonstrate that ROS content in DPSC and MC3T3-E1 cells that stuck to the surface of titanium and zirconium after 24 h of incubation is high enough to allow for the correct process of cell adhesion and spreading. These processes are more efficient for the zirconia surface [47]. Pietropaoli et al. [47] believe that both the excessive production of ROS and, consequently, AGEs in the tissues adjacent to the implant are factors that favor the implant rejection process. In the opinion of these authors, ROS-derived AGEs that irreversibly accumulate in the tissues surrounding the implant, disturb the collagen structure and induce inflammation. However, the redox imbalance in the course of peri-implantitis is less severe than in periodontal diseases. Similar results of redox equilibrium changes were obtained by Guo et al. [54]. but it should be emphasized that these observations were found in patients’ saliva. Cabaña-Muñoz et al. observed that patients with long-term (10 years) dental titanium and amalgams have systemic oxidative stress expressed as a significant increase in MDA concentration and decrease in Mo/Co and Mo/Fe^2+^ ratios in hair samples than those with amalgam alone [119]. On the other hand, Sánchez-Siles et al. [120] found that salivary concentration of myeloperoxidase and MDA in patients with peri-implantitis and without periodontal diseases did not differ from the healthy control. According to the results of Mousavi et al. [55], there is a positive correlation between periodontal pocket depth (PPD) around the implant and MDA and TAC concentrations. However, the concentration of the tested OS markers (MDA, SOD, TAC) in an osmotically mediated transudate/inflammatory exudate around dental implants (PICF) in diseased implants (PPD ≥ 4 mm, gingival index GI ≥ 1, bleeding on probing BOP = 1) did not differ from “healthy implants” (PPD < 3 mm, GI = 0, BOP = 0). According to the authors, the study on the aforementioned OS markers does not allow for the differentiation between peri-implant health and disease conditions.

As a result of processes such as friction, titanium implants contribute to the increased production of ROS and increase the carbonyl, MDA, AGE proteins in the tissue around the implant. These observations call into question the safety of leaving titanium implants permanently.

## 12. Periodontology

Periodontal diseases are a very common problem in dental practice. Interestingly, a key role in periodontitis is played by oxidative stress [121,122]. In response to bacterial microflora and local inflammation, polymorphonuclear leukocytes (but also peripheral blood leukocytes) produce large amounts of ROS, which by oxidizing lipids, proteins, and nucleic acids, lead to the destruction of periodontal connective tissue. Thus, periodontitis may lead to increased tooth mobility and even tooth loss [52].

A question arises: can periodontal treatment influence the intensity of salivary oxidative stress? Tamaki et al. [53] demonstrated that scaling and root planning (SRP) significantly reduced the level of reactive oxygen metabolites (ROMs) in the plasma of patients with chronic periodontitis. Moreover, the rate of ROMs production correlated significantly with the severity of periodontal disease (a positive correlation with bleeding on probing [BOP] and clinical attachment loss [CAL]) both before and after the surgery [53]. SRP also considerably reduced the concentration of salivary malondialdehyde (MDA), which may—to some extent—explain the therapeutic success in patients with non-surgical periodontal treatment [53].

The salivary content of GSH is significantly lower in the saliva of patients with chronic periodontitis [52,123], which is not surprising as this compound is used intensively by periodontal tissues in response to increased ROS production. However, SRP together with 7-day antibiotic therapy (amoxicillin + metronidazole, 500 mg each, 3 times daily) does not change salivary levels of GSH or C-reactive protein (CRP) in patients with periodontitis [52]. Boia et al. [52] demonstrated that non-surgical periodontal therapy lowered ROMs levels in plasma. This parameter correlates positively with CAL, which confirms previous reports on the relationship between ROS production in blood and periodontal inflammation [52,53].

Recently, antimicrobial photodynamic therapy (aPDT) has become a popular method of treatment of periodontitis patients. The term aPDT means a laser therapy in which the photosensitizer produces oxygen free radicals that destroy microorganisms in the gingival pocket biofilm [51]. Indeed, it was demonstrated that laser light in the presence of methylene blue (MB) in 20% ethanol generates significant amounts of ROS, which determines its bactericidal effect. Pillusky et al. [51] also showed that aPDT increases the concentration of erythrocytic GSH, which is the systemic adaptive response to the applied treatment. Photodynamic therapy can be performed as an additional procedure to standard periodontal treatment to enhance the systemic protective response against oxidative stress, boosting and accelerating periodontium healing, particularly when the photosensitizer is dissolved in ethanol (which facilitates its penetration into periodontal tissues) [51].

Ozone (O_3_) is also very popular in periodontology. It was demonstrated that O_3_ increases ROS production in periodontal tissues, thus leading to the activation of numerous transcription factors such as mitogen-activated protein kinase (MAPK), cellular proto-oncogene encoding the transcription factor FOS (c-Fos), AP-1 (transcription factor activator protein 1) and the nuclear factor erythroid 2 (NF-E2)-related factor 2 (Nrf2). Importantly, Nrf2 is related to the promoter region of metallothionein that participates in the response to OS [56]. Leewananthawet et al. [56] proved that aqueous ozone ultrafine bubble water (OUFBW) increases the antioxidant defense of periodontal ligament fibroblasts, while the stimulation of c-Fos and c-Jun pathways further differentiates osteoblasts involved in periodontal tissue regeneration [56]. A study on an animal model showed that blue light increases the amount of ROS, which results in vasospasm in the aorta. Moreover, researchers suggest that the elevated ROS level under the influence of blue light also occurs in gingival fibroblasts. The alternating vasospasms and vasodilatations due to ROS lead to the subsequent generation of reactive oxygen species, which is called the vicious circle effect [124,125].

The ROS generated in the treatment of periodontal diseases seems to positively affect the healing process: they support the elimination of periopathogenic bacteria and accelerate periodontium healing.

## 13. Whitening

Tooth whitening is one of the most frequently chosen procedures to restore the esthetics of discolored teeth. Whitening can be performed both in the dental clinic and at home [126,127]. Hydrogen peroxide (H_2_O_2)_ is the most commonly used substance for this purpose due to its low price and high effectiveness [83]. H_2_O_2_ is characterized by high reactivity, although it is not a free radical. It participates in oxidation reactions, the most biologically significant of which is the oxidation of sulfhydryl groups. [128]. It leads to the formation of disulfide bridges, which changes the conformations as well as biological functions of proteins. H_2_O_2_ also has the ability to oxidize unsaturated fatty acids during the process of lipid peroxidation. [129]. Moreover, hydrogen peroxide is capable of oxidizing ferrous or copper ions (the proper Fenton reaction: Fe^2+^ + H_2_O_2_→Fe^3+^ + ·OH + OH^−^, the catalysts for this reaction are also Cu^2+^ ions). During these reactions, hydrogen peroxide is transformed into hydroxyl radical ·OH. The redox reaction under the influence of H_2_O_2_ results in the formation of: superoxide anion, hydroxyl radical and singlet oxygen, all of which have an oxidizing effect on the organic components of the enamel, leading to the whitening of tissues [67,72]. It is suggested that low concentrations of hydrogen peroxide do not exhibit a destructive effect on the oral cavity cells [125]. ROS generated during the whitening process induce a sequence of reactions that increase the expression of heme oxygenase 1 (HO-1) in fibroblasts and osteoblasts [83]. Considering the antioxidant and anti-inflammatory properties of this enzyme, it can be concluded that increased expression of HO-1 in cells exposed to H_2_O_2_ is a defensive mechanism against the adverse effects of whitening.

Del Real Garcia et al. [57] demonstrated that in patients exposed to 10% H_2_O_2_ contained in whitening strips (Crest^®^ 3D Whitestrips^®^ premium plus), the concentration of salivary 8-OHdG increased compared to non-exposed subjects. Moreover, the positive correlation between the genotoxicity test and 8-OHdG concentration indicates that hydrogen peroxide is the cause of DNA damage in patients using whitening strips. Indeed, in the study group the authors observed chromosome damage, impaired cytokinesis and increased apoptosis of the oral epithelial cells after 15 and 30 days of using whitening strips [57]. Interestingly, no DNA damage was demonstrated after professional whitening treatments with 35% hydrogen peroxide. Whitening procedures are only suggested when performed by qualified dentists [57].

Interestingly, hydrogen peroxide can diffuse through the enamel to a concentration of about 0.01% in the pulp. Recent studies suggested that the higher the content of whitening agents such as hydrogen peroxide, the greater the cytotoxic effect on the pulp [127]. Cytotoxicity increases significantly after using a LED lamp with a wavelength from 400 to 505 nm. In the study group using 0.01% hydrogen peroxide, a considerable increase in ROS production as well as boosted apoptosis on the mitochondrial pathway were demonstrated, which was associated with a decreased number of living cells in this group [127]. However, it was shown that the pulp after whitening undergoes regeneration within 24 to 72 h, which indicated a temporary cytotoxic effect [127]. Lima et al. [130] also demonstrated that the use of antioxidants such as sodium ascorbate (0.25 mM/0.5 mM) reduces ROS production, thus limiting the risk of OS development (85). Vargas et al. observed that the use of alpha-tocopherol (1, 3, 5 and 10 mM) reduced hydrogen peroxide-induced cytotoxicity (0.035%, 0.018%, 0.009% and 0.045%) in odontoblasts and pulp cells [131].

The use of appropriate concentrations of bleaching agents generates ROS while simultaneously induces antioxidant defense systems. Consequently, the whitening process takes place without adverse effects on the redox equilibrium of the oral cavity.

## 14. Summary

As a result of OS, cell components—mainly proteins, lipids and nucleic acids—are damaged, which leads not only to structural changes of the cell, but also to its death through apoptosis and necrosis. Moreover, oxidative stress is associated with the pathogenesis of numerous systemic diseases, including those related to the oral cavity. Interestingly, not only the diseases of the oral cavity, but also their treatment induce OS. The source of ROS in the oral cavity can be filling materials such as amalgam, composites, glass-ionomer materials, or bonding systems, but also agents used in endodontic treatment, periodontal and surgical procedures, or materials used in orthodontic treatment.

Although some of the listed ROS/RNS sources in the oral cavity are inevitable and, in some situations, beneficial (periodontal treatment), the search for therapeutic solutions to avoid materials and treatment procedures leading to ROS overproduction seems extremely important.

## Figures and Tables

**Table 1:** Research included in the study.

Experimental Model	Endpoints	References
Amalgam		
Human gingival fibroblast cells (HGFCs) exposed to microhybrid resin-based composite, compomer resin, glass-ionomer cement and amalgam alloy for 7 and 21 days	The levels of total oxidant status (TOS) in the study groups (i.e., samples with the following materials: microhybrid resin-based composite, compomer resin, glass-ionomer cement and amalgam alloy; shaped as a 2-mm-thick disk with a diameter of 10 mm; exposed to light with the wavelength of 430–480 nm and intensity of 1200 mW/cm^2^) were significantly higher in freshly prepared samples compared to the control. After 7 and 21 days, TOS level in the amalgam sample was considerably lower than at the beginning of the study. The highest level of total antioxidant capacity (TAC) was observed after 7 days in the filling with glass-ionomer cement (which prevented TOS increase). In all studied groups, TAC level after 7 days was different than at the initial stage of the study.	[19]
Unstimulated saliva of 48 generally healthy children aged 6–10 (24 males, 24 females) with two class II dental composite or amalgam restorations and the control (caries-free) group	The saliva of patients with composite fillings had significantly higher TAC compared to patients with amalgam fillings as well as caries-free subjects. However, TAC in patients with amalgam restorations was also significantly higher compared to the caries-free control. Patients with composite fillings also demonstrated decreased salivary levels of Ca^2+^ ions.	[20]
Saliva from 60 generally healthy subjects aged 15–40 with class I restorations of: amalgam (20 participants), composite (20 subjects) and glass-ionomer (20 patients), collected before the filling as well as 24 h and 7 and 14 days after the filling	Malondialdehyde (MDA) level in the saliva of patients with an amalgam filling was found to be higher than in patients with a composite or glass-ionomer filling. Significant differences were also observed between MDA concentrations on day 7 and 14, and after 24 h and 7 days in patients with composite fillings. There were no differences in MDA levels before treatment and 7 days after, or before and 14 days after the treatment. In the case of glass-ionomer, a significant difference was found only between 24 h and 7 days after the treatment.	[21]
Urine collected from 106 generally healthy children aged 5–15.5 years with amalgam fillings	It was shown that in children with amalgam filling, there was a reduced excretion of 8-hydroxy-2-deoxyguanosine (8-OHdG) in the urine. It was also shown that the level of NAG in the urine of children with amalgam fillings was significantly higher compared to children without such fillings and was positively correlated with the level of MDA in the urine. There was no correlation between the concentration of 8-OHdG and malondialdehyde (MDA) in the urine of amalgam-filled children. The mercury (Hg) level was also significantly higher in children with amalgam fillings compared to children without amalgam fillings; however, no relationship was found between the Hg level and the number of fillings.	[22]
Hair samples collected from 42 generally healthy women (mean age 44 years) with amalgam fillings applied at least 10 years earlier	An increased activity of SOD-1 and an increase in GSH concentration in the hair of women with amalgam fillings as compared to women without such fillings were observed. A positive correlation was also shown between the concentration of aluminum (Al) and the concentration of GSH, and between the level of mercury (Hg) and the activity of SOD-1.	[23]
Blood collected from 41 generally healthy patients (17–23 years old), which used amalgam (19) and dental resin composite (22) fillings	A significant increase in the level of malondialdehyde (MDA) was observed 24 h after placing amalgam and composite filling. There were no changes in the concentration of 8-OHdG in women 24 h after the placement of the amalgam filling. The 8-OHdG level increased 24 h after placing the dental resin composite filling.	[24]
Dental Resin Composites—monomers		
Human dental pulp cells (hDPCs) exposed to dental monomers (1 mM HEMA, 5 mM MMA and 1 mM TEGDMA) without and in the presence of 10 mM NAC for 24, 48, 72 and 96 h	In response to 6 h of exposure to dental monomers: 2-hydroxyethyl methacrylate (HEMA), triethylene glycol dimethacrylate (TEGDMA) and methyl methacrylate (MMA), there was a significant increase in ROS production in hDPCs compared to the control group without dental monomers. The addition of N-acetyl cysteine (NAC) decreased ROS production in the monomer-treated group. The presence of monomers also GSH level, which was observed for NAC as well, but to a lesser extent. No significant differences in the content of GSSG (oxidized disulfide) were observed for HEMA and MMA monomers, and a slight GSSG decrease was noted for TEGDMA (triethylene glycol dimethacrylate). In the case of dental monomers, MDA level increased, and after adding NAC—MDA level dropped almost to its level observed in the control group. Moreover, SOD activity decreased in the presence of all dental monomers, which was not observed after the addition of NAC. After 24 h of cell exposure to monomers, CAT activity increased significantly, and decreased after the use of NAC.	[25]
Human dental pulp cells isolated form third molars, exposed to dental monomers (bisphenol-A-glycidyl Methacrylate, Bis-GMA; urethane dimethacrylate, UDMA; and triethylene glycol dimethacrylate, TEGDMA) at concentrations of 10, 30, 100, 300 µm for 48 h	The level of free radicals was measured after 48 h of monomer action by means of 2′,7′-dichlorodihydrofluorescein diacetate (DCF) fluorescent dye, and it was observed that Bis-GMA and UDMA, at high concentrations (30, 100), induced a significant increase in oxidative stress, while the TEGDMA monomer did not trigger OS at any concentration. All monomers reduced the level of GSH.	[26]
Smulow-Glickman (S-G) human gingival epithelial cells and pulp fibroblasts (HPF) exposed to HEMA at the concentrations of 0.01–10 mm for 24 h	Higher HEMA concentrations (1, 2.5, 5, 10) caused a significant increase in the level of intracellular ROS in cells exposed to the monomer.	[27]
Gingival fibroblasts obtained during the extraction of premolars for orthodontic reasons, exposed to TEGDMA at a concentration of 0.6 mM and 1 mM for 15 min to 6 h	15-min exposure to TEGDMA significantly reduced the concentration of intracellular GSH compared to cells not exposed to this monomer. It was also demonstrated that TEGDMA-induced time-dependent increase of thiobarbituric acid reactive substances (TBARS), which indicates increased lipid peroxidation.	[28]
Human dental pulp stem cells (isolated from third molars) exposed to monomer HEMA (at a concentration of 2 mM) and AC (at 50 µg·mL^−1^) for 24 h	2-hydroxyethyl methacrylate (HEMA) increased the level of reactive oxygen species (ROS), pro-inflammatory mediators such as nuclear factor-κB (NF-kB) and inflammatory cytokines such as interleukin. In the presence of vitamin C, these changes were less noticeable. This indicates a protective effect of vitamin C on the dental pulp cells.	[29]
Human gingival fibroblasts (HGFs) treated with a relatively low level of 2-hydroxyethyl methacrylate (HEMA) for 0, 24 and 96 h	After 24 and 96 h of HGF exposure to the HEMA monomer (3 mmol·L^−1^), it was observed that ROS levels increased 8 and 11 times compared to the control not exposed to the monomer.	[30]
Primary human gingival fibroblasts (HGFs) and immortalized oral keratinocyte cell line OKF6/TERT2 treated with 2-hydroxyethyl methacrylate (HEMA) at a concentration of 0.5–10 mM	Significantly induced transcription of genes related to defense against oxidative stress was demonstrated for: nuclear factor erythroid 2-related factor 2 (Nrf2), heme oxygenase (HO-1), quinone dehydrogenase 1 (NQO1), superoxide dismutase 1 (SOD1) in both cell types exposed to the HEMA monomer. The transcription of nuclear factor kappa-light-chain-enhancer of activated B cells (NF-κB) and interleukin-6 (IL-6) was repressed in both cell types, while the transcription of tumor necrosis factor α (TNF-α) and interleukin-8 (IL-8) was repressed only in OKF6/TERT-2 cells.	[31]
Primary human dental pulp cells (hDPCs) obtained from healthy patients aged 18–25, during the extraction of healthy third molars, exposed to 1 mM 2-hydroxyethyl methacrylate (HEMA) for 18 and 12 h	It was demonstrated that the expression of NFE2L2 (nuclear factor, erythroid 2 like 2) and HMOX1 (heme oxygenase (decycling) 1) genes encoding the proteins: Nrf2 (nuclear factor erythroid 2-related factor 2) and HO-1 (heme oxygenase 1) in the HEMA-exposed group increased compared to the group not exposed to HEMA.	[32]
Human gingival fibroblasts (HGFs) exposed to 2-hydroxyethyl methacrylate (HEMA) and triethylene glycol dimethacrylate (TEGDMA) at a concentration of 3 mM for 24, 48 and 72 h	It was demonstrated that exposure to HEMA caused autophagy and apoptosis in each of the analyzed periods of time. No signs of autophagia were observed in TEGDMA-exposed cells	[33]
	Dental Resin Composites—Cross-linked samples	
Human dental pulp cells exposed to methacrylate-based dental resin composite, including triethylene glycol dimethacrylate and composites free of 2-hydroxyethyl methacrylate and silorane-based composite (5 mm in diameter and 2 mm high) cured with light (780 mW/cm^2^) for 40 s in the presence of dental polymers (reduction of free radical polymerization) and absence of polyester film	Flow cytometry showed increased ROS production in cells exposed to dental resin composite materials. A positive correlation was observed between ROS production and cell survival in groups not covered with polyester film. TEGDMA increases ROS production.	[34]
Human dental pulp cells (isolated from third molars) exposed to dental material dental resin composite s for 48 h with IGF-1 i TGF-b	Insulin-like growth factor (IGF-1) and transforming growth factor beta (TGF-β) increased cystine capture, resulting in elevated levels of cellular glutathione in a group of cells exposed to dental resin composite (Flow Line, 9.5 +/− 0.4 mg and Durafill VS, 10.0 +/− 0.4 mg). This provided increased protection against OS effect triggered by dental resin composite.	[35]
Human pulp cells obtained from impacted third molars, exposed to cured bonding agents (Clearfil SE Bond, CB; Prime & Bond 2.1, PB; and Single Bond, SB) at a concentration of 10 µL for 2 days	Dentine bonding agents decrease the level of GSH, which might be the reason for the cytotoxicity of resins. Cytotoxicity decreased when N-acetyl-L-cysteine (NAC) was added to the sample.	[36]
Saliva collected from 52 patients (32 women and 20 men) who had been treated with Filtek Z250 dental resin composite fillings, before the filling and 1 h, 1 day, 7 and 30 days after the filling	Patients with dental resin composite fillings demonstrated a significantly increased MDA level compared to subjects without fillings, but there were no statistical differences between the studied time periods. There was also a significant decrease in SOD activity 7 days after the filling compared to the controls. No significant differences were noted in SOD values between day 7 and 30 in patients with dental resin composite fillings.	[37]
Composite resins		
Human gingival fibroblasts (HGFs) exposed to composite resin (consisting of 45% 2-hydroxyethyl methacrylate—HEMA and 55% bisphenol A-glycidyl dimethacrylate—Bis-GMA) at concentrations of up to 0.25 mM	It was demonstrated that the expression of 8-hydroxyguanine in DNA– hydrolase I, the main enzyme for repairing 8-oxoG damage in composite resin-exposed cells, was elevated compared to cells not exposed to monomers.	[38]
Mouse fibroblast cells (NIH/3T3) exposed to camphorquinone (CQ), CQ and diphenyleneiodonium hexafluorophosphate (DPI), CQ and ethyl 4-dimethylamino benzoate (EDAB), and CQ, EDAB and DPI, with EDAB in high and low concentration, for 10 and 20 s	Increased activity of SOD was observed after 10 s of polymerization vs 20 s in NIH/3T3.	[39]
Orthodontic braces		
L929 mouse fibroblast cell line exposed to six types of orthodontic archiwires (stainless steel, nickel-titanium, copper-nickel-titanium, rhodium-coated nickel-titanium, cobalt-chromium Blue Elgiloy, titanium-molybdenum) in 1-cm-long pieces (1 mL saliva per 0.2 g of the wire)	It was demonstrated that a standard nickel-titanium orthodontic archiwire generates the strongest oxidative stress, while stainless steel and titanium-molybdenum wire triggers the lowest OS in a mouse fibroblast cell culture.	[40]
L929 mouse fibroblast cell line exposed to three conventional (stainless steel, monocrystalline sapphire ceramics, polyurethane) and four self-ligating brackets (stainless steel body with a nickel-titanium clip, aluminum oxide ceramics with a cobalt-chromium clip, aluminum oxide ceramics with a nickel-cobalt clip coated with rhodium, polycarbonate-stainless steel brackets) made of different materials	The assessment of 8-hydroxy-29-deoxyguanosine (8-OHdG) in DNA of L929 murine fibroblast cell line demonstrated that the lowest OS is triggered by a conventional sapphire ceramic bracket. Full metal conventional and self-ligating brackets and conventional polyurethane brackets showed higher OS compared to cells not exposed to these brackets. The highest OS is caused by full metal and polyurethane brackets.	[41]
Saliva of 23 patients aged 12–16 enrolled in the study (12 female, 11 male subjects), treated with multibracket self-ligating vestibular orthodontic appliances	During the first 10 weeks of treatment with multibracket self-ligating vestibular orthodontic appliances, no statistically significant changes in the salivary antioxidant test (SAT) were observed.	[42]
Unstimulated saliva and gingival fluid of 50 generally healthy patients (27 females and 23 males) aged 13–20, treated with permanent brackets, collected before the treatment as well as in the 1st and 6th month of the treatment	There was no increase in oxidative damage (8-OHdG, MDA) in the saliva and gingival fluid of patients treated with permanent brackets compared to pre-treatment results.	[43]
Unstimulated (UWS) and stimulated (SWS) saliva of 37 generally healthy subjects treated with permanent orthodontic brackets, collected immediately after the fitting of the brackets as well as 1 week and 24 weeks after the fitting	There was a significant increase in thiobarbituric acid reactive substance (TBARS) in UWS and SWS one week after braces were fitted. The measured values returned to their initial state 24 weeks after the beginning of the treatment. There were no significant differences between the levels of SOD1, CAT, UA and Px activity in UWS 1 week and 24 weeks after the start of treatment. SOD1 activity was found to be significantly lower in SWS, and Px activity was considerably higher 1 week after the placement of the brackets compared to the values before the treatment and 24 weeks after its commencement. The total antioxidant status (TAS) in UWS and SWS was also found to be considerably lower 24 weeks after the start of the treatment compared to the values before the treatment as well as 1 week after its start. The highest oxidative stress index (OSI) values were observed 1 week after the treatment. 24 weeks after the treatment these values were identical to pre-treatment results.	[44]
Fixations and dental implants		
Human dental pulp stem cells (DPSC) and murine pre- osteoblast (MC3T3-E1) cells exposed to zirconium and titanium oxide for 24 h	Intracellular oxidation of 5-(and -6)- chloromethyl-2′,7′-dichlorodihydrofluorescein diacetate and acetyl ester (CM-H2DCFDA), a ROS indicator dye, demonstrated relatively higher average ROS levels in both types of cells exposed to zirconium compared to titanium.	[45]
Periosteum of 30 patients (8 women and 22 men) with bilateral fractures of the mandible, treated with Ti6Al4V titanium alloy	The periosteum of patients treated with titanium implants showed significantly higher concentrations of the biomarkers of nitrosative (S-nitrosothiols, peroxynitrite, nitrotyrosine) and oxidative stress (malondialdehyde, protein carbonyls, dityrosine, kynurenine and N-formylkynurenine) compared to the control without titanium fixations. Osteosynthesis patients also demonstrated increased antioxidant protection expressed in elevated levels of reduced glutathione (↑GSH) and glutathione reductase (↑GR). The periosteum of patients with titanium fixations revealed a considerable decrease in the activity of mitochondrial complex I (−77.8%) and CS (citrate synthase) (−166.7%) compared to the control. There were no statistically significant differences in the activity of complex II and cytochrome C oxidase (COX) between patients after osteosynthesis as compared to healthy controls. In the periosteum of osteosynthesis patients, the production of hydrogen peroxide as well as the rate of ROS production were also significantly increased. Titanium implants caused oxidative/nitrosative stress and mitochondrial dysfunction. Moreover, a positive correlation between ROS production rate and GSH concentration was observed, which may suggest increased antioxidant defense in patients after osteosynthesis.	[46]
Whole saliva of patients aged 43–57 with peri-implantitis and five titanium implants (collected from five patients) that were rejected up to 6 months after their implantation (3 from the mandible, 2 from the maxilla); oxidative stress parameters	In the course of peri-implantitis, a significant increase was observed in AGE compared to the control. In the saliva of peri-implantitis patients the level of OS was higher than in healthy individuals.	[47]
Periosteum of 32 patients operated on due to class III dentofacial deformities (21 women and 11 men aged 20–30), who had had titanium implants inserted and then removed 12–30 months after the implantation	Decreased activity of superoxide dismutase-1 (SOD1) (↓37%) and tryptophan level (↓34%) as well as significantly higher content of advanced oxidation protein products (AOPP) (↑25%), total oxidant status (TOS) (↑80%) and oxidative stress index (OSI) (↑101%) were observed in the maxillary periosteum of osteotomized patients compared to the controls. The mandibular periosteum demonstrated a significant decrease in SOD-1 activity (↓55%), total oxidant status (TAC) (↓58.6%), advanced glycation end products (AGE) (↓60%) and N-formylkynurenine (↓34%), and considerably increased content of AOPP (↑38%), malondialdehyde (MDA) (↑29%), 4-hydroxynonenal (4-HNE) (↑114%), TOS (↑99%) and OSI (↑381%) compared to the controls. Further weakening of the redox economy and increased ROS production were demonstrated in the mandibular periosteum compared to the maxillary periosteum.	[48]
Periosteum of 29 patients (aged 19–29) treated with titanium implants (due to a bilateral mandibular shaft fracture) that were removed 3–5 months after the procedure	The periosteum of patients after osteosynthesis showed significantly higher activity of NADPH and xanthine oxidase, and increased rate of free radical production compared to the control. The periosteum of patients after osteosynthesis also demonstrated a considerable increase in the levels of inflammation markers: interleukin 1 (IL-1), interleukin 6 (IL-6), tumor necrosis factor α (TNF-α), transforming growth factor β (TGF-β) and β-glucuronidase (GLU) as well as markers of apoptosis (Bax, Bax/Bcl-2), caspase-3 (CAS-3) and nitric oxide (NO) compared to the control. Titanium implants increased the production of proinflammatory cytokines and oxygen free radicals. A positive correlation between titanium content and CAS-3 activity was also demonstrated.	[49]
Periosteum, plasma, and erythrocytes collected from 31 generally healthy subjects aged 21–29 (11 women and 20 men) with bilateral mandibular fractures treated with titanium miniplates (Ti4Al4V)	Decreased CAT activity in the mandibular periosteum and its increase in erythrocytes of patients with mandibular fracture treated with titanium miniplates were demonstrated compared to the subjects not exposed to titanium implants. SOD activity and UA concentration were significantly higher in both plasma and periosteum of fracture patients compared to healthy individuals. No differences were found in GPx activity between the studied groups. There was an increase in TAC, FRAP, TOS, AGE, AOPP, 4-HNE and a decrease in OSI level in the maxillary periosteum of patients with fracture compared to healthy subjects. There were no significant differences in plasma TAC, TOS, OSI, FRAP AGE, AOPP, 4-HNE and 8-OHdG levels between patients with a fracture and healthy subjects. A positive correlation was observed between TAC concentration in the mandibular periosteum and plasma UA level in patients with a mandibular fracture. A positive correlation was also found between TOS concentration in the periosteum and CAT activity in erythrocytes, and between 8-OHdG level in the periosteum and GPx activity in erythrocytes.	[50]
Periodontology		
Male adult Wistar rats (2 months of age) with periodontitis, subjected to antimicrobial photodynamic therapy (aPDT)	PDT was shown to increase ROS formation as well as boost the antioxidant response.	[51]
Whole saliva of patients aged 43–57 with peri-implantitis and 5 titanium implants (collected from five patients), which were rejected up to 6 months after their implantation (3 from the mandible, 2 from the maxilla)	In patients with peri-implantitis, the western blot technique revealed a significant increase in AGE compared to healthy controls. By means of TBARS assays, a higher level of OS was also observed in the saliva of peri-implantitis patients compared to healthy subjects.	[47]
Sixteen patients with chronic periodontitis (CP), undergoing non-surgical periodontal therapy alone as well as non-surgical therapy accompanied by antibiotic therapy of Amoxicillin + Metronidazole, 500 mg each, 3 times daily, for 7 days	It was demonstrated that after 3 months OS levels decreased from very high to average during antibiotic therapy, as shown by reduced derivatives of reactive oxygen metabolites (d-ROMs) (from 491.83 ± 134.85 U CARR to 375.58 ± 126.06 U CARR) and reduced glutathione (GSH) (from 48.73 ± 33.89 μmol/L to 46.46 ± 21.59 μmol/L) in plasma.	[52]
Nineteen patients with chronic periodontitis (average age: 46.8 years) examined before the therapy (scaling and root planing) as well as 1 and 2 months after the therapy.	Non-surgical treatment of periodontitis reduced plasma ROM levels compared to pre-treatment levels.	[53]
Tissues and saliva of 10 patients with peri-implantitis and 10 with chronic periodontitis, aged 40–60	In both the saliva and tissues of patients with peri-implantitis and chronic periodontitis, AGE levels more than doubled compared to healthy individuals. A strong positive correlation was also observed between ROS and AGE in the examined patients.	[54]
Peri-implant crevicular fluid (PICF) collected from 31 patients	The concentration of MDA, SOD and TAC in peri-implant crevicular fluid did not differ from that in healthy subjects. However, there was a positive correlation between periodontal pocket depth (PPD) around the implant and MDA and TAC levels.	[55]
Whitening		
Human primary periodontal ligament fibroblasts (hPDLFs) and Ca9-22 human gingival epithelial cells treated with stable aqueous ozone ultrafine bubble water (OUFBW; ozone concentration: 2.5 ppm) or UV-inactivated OUFBW	OUFBW (30 min of incubation) stimulated ROS production in both cell lines, thus activating the MAPK pathway. OUFBW triggered the activation of c-Fos, a major component of the transcription factor activator protein 1 (AP-1), and also nuclear factor erythroid 2 (NF-E2)-related factor 2 (Nrf2), which demonstrated high sensitivity to oxidative stress.	[56]
One-hundred thirteen patients (60 people using Crest^®^ 3D Whitestrips^®^ premium plus, 10% hydrogen peroxide, 53 subjects in the control group). Oral epithelial cells and saliva samples were collected at the beginning of the study and 30 days later from the control group, and immediately before whitening as well as 15 and 30 days after the completion of the whitening procedure	After the whitening procedure, an elevated level of 8-OHdG in saliva and a positive correlation between oxidative stress produced by hydrogen peroxide and micronuclei were found.	[57]
